# Impact of various drying technologies for evaluation of drying kinetics, energy consumption, physical and bioactive properties of Rose flower

**DOI:** 10.1038/s41598-025-94300-x

**Published:** 2025-03-18

**Authors:** Mohammad Kaveh, Shahin Zomorodi, Behnam Ghaysari, Hany S. El-Mesery, Faroogh Sharifian, Ahmed H. ElMesiry, Ali Salem

**Affiliations:** 1 Agricultural Engineering Research Department, West Azerbaijan Agricultural and Natural Resources Research and Education Center, AREEO, Urmia, Iran; 2 Department of Horticulture, Faculty of Agriculture, Urmia University, Urmia, Iran; 3 School of Energy and Power Engineering, Jiangsu University, Zhenjiang, 212013 People’s Republic of China; 4 Agricultural Engineering Research Institute, Agricultural Research Center, Dokki, 12611 Giza Egypt; 5 Department of Mechanical Engineering of Biosystems, Faculty of Agriculture, Urmia University, Urmia, Iran; 6 Faculty of Computer Science and Engineering, New Mansoura University, Mansoura, 35742 Egypt; 7 Civil Engineering Department, Faculty of Engineering, Minia University, Minia 61111, Egypt; 8 Structural Diagnostics and Analysis Research Group, Faculty of Engineering and Information Technology, University of Pécs, Pécs 7622, Hungary

**Keywords:** Rose flower, Refractance window, Drying, Antioxidant activity, Total phenol, Plant sciences, Plant reproduction, Energy infrastructure

## Abstract

The process after harvesting medicinal plants, such as drying, is very important in the production cycle of these plants. The study’s objective is to evaluate the effect of different drying methods on some thermodynamic properties, qualitative and bioactive attributes, and yield of rose essential oil in form of a completely randomized design. The treatments of this study included drying in refractance window (RW), infrared (IR), and convective (CV) at three drying temperatures of 50, 60, and 70 °C, as well as fresh plants. The results showed that different drying methods and temperatures significantly affected the essential oil, thermodynamic, qualitative, bioactive, and yield characteristics. The lowest drying time, energy consumption, and the highest energy efficiency and rehydration ratio in the dried rose samples were related to the drying temperature of 70 °C in the RW method. The reduction of drying time by RW method compared to IR and CV methods was between 11.1–21.40 and 45.9–50%, respectively. The highest amount of antioxidant activity, total phenol, flavonoid and essential oil yield was observed in the RW drying method and at the drying temperature of 60 °C. This study showed that compared to other drying methods, the RW method showed a high quality in drying Rose flowers.

## Introduction

Roses are shrubs of the Rosaceae family. This family includes over 2000 species and about 100 genera^1^. Rose has long been popular with many people due to its many properties, such as its relaxing and energizing qualities. Rose flower essential oil is also one of the most valuable essential oils and is classified as the third essential oil in terms of volume value^2^. Its uses are in the perfumery and aromatic industries, health-cosmetic products, food industries, including all kinds of sweets, drinks, jelly, etc., and pharmaceutical industries^3^. Due to the limited harvest season, the perishability and the rapid reduction of the effective substance of the rose essential oil due to the late delivery of the harvested flowers to the factory, the inability to preserve the flowers, and the high rate of spoilage in the products that were harvested wet, it seems necessary to take actions to prevent the emergence and spread of mold^4^. Drying agricultural products causes a lack of the necessary environment for the growth of living organisms such as yeasts, molds, and bacteria^5^. Drying is one of the important processes in the processing of rose flowers, and it has many applications in the pharmaceutical, food, and perfumery industries. Therefore, it seems necessary to design an operational system for the drying process.

Choosing the right method for drying plant organs is among the most important in post-harvest operations. Using an inappropriate method can lead to the destruction of plant organs or the destruction of all the effective substances in them. Since there are different generations of traditional and new drying technologies, choosing a suitable drying method for the desired products is always challenging for the food industry^6^. The natural drying method (shade and sun) has many disadvantages, such as the impossibility of moving large amounts of plant matter, achieving consistent quality standards, limited sunlight time, and a short harvesting season^7^. Disadvantages of the hot air-drying method include low energy efficiency and a time-consuming process^8,9^. Drying with RW is one of the new and low-consumption methods of the fourth generation of dryers used to dry heat-sensitive products such as medicinal plants^10,11^. In the RW technology, hot water circulating in a shallow tank is used as a heating medium, where the food material is placed on a flexible polyester film (Mylar™) that is in contact with the heating medium^12,13^. In this process, the thermal energy of the heating environment that circulates through the tank is transferred to the food through conduction and radiation through the flexible polyester film^14^. The short time used, high thermal efficiency, preserving the color of dried plants, improving the content of active ingredients and preserving vitamins and antioxidants are among the important advantages of this technique. Several studies have tried to use product quality as a measure to evaluate different drying technologies^15^. Similarly, the impact of four drying methods (RW, vacuum-RW, CV and oven drying) on color and texture characteristics of Tarhana dough was reported^13^. The effects of drying with RW, freezing, vacuum, and CV on phytochemical characteristics and physical quality of banana slice and pulp were evaluated by Dadhaneeya et al.^11^. Goldenberry drying was done using CV, IR, RW, and freezing methods, and their effects on color changes, blue activity, and antioxidant components were investigated^16^. Zamani et al.^17^ studied the physical, bioactive and synthetic properties of Dracocephalum kotschyi drying in RW, IR-RW, CV, shade and solar methods. On the other hand, studies of drying with RW compared to different dryers on aonla^18^, spent coffee grounds^19^, beetroot^20^, onion^21^, Maraş green pepper^22^ and physalis^23^ have been reported. Compared to conventional drying methods, these studies showed that the product dried with RW has better quality in a shorter time and with minimum energy consumption. Studies have also shown that this method is more suitable for drying thin-layer materials while increasing the thickness reduces its efficiency.

Most previous studies on drying evaluation used a single product (fruit, vegetable or medicinal plant) to compare the qualitative aspects of the dried sample. As a result, the findings of these studies mainly indicate the maintenance of product quality for the selected product, which may not be generalizable to other products. Also, to date there is no information on the evaluation of energy efficiency, thermodynamic parameters and essential oil yield using RW dryers for medicinal plants. Few reported works have been done on RW dryers for other agricultural products. Therefore, these results can be compared with those of a traditional dryer such as a hot air and infrared dryer not only to analyze energy savings but also other food properties that are not considered. Also, no comparative work has been done on medicinal plants with RW or hot air and infrared dryer. Finally, the preservation of bioactive substances, and physical properties and essential oil yield in rose flower have not been reported. Therefore, the main goal of this comprehensive study is to investigate the effect of three drying methods (refractance window, infrared, and hot air) on Rose flowers’ thermodynamic, physical, qualitative, bioactive, and microstructural properties. Choosing the right drying method can provide a better understanding of how researchers can produce high-quality medicinal plants and reduce post-harvest losses.

## Materials and methods

### Test samples

In this research, Rose flowers were randomly picked from a farm in Urmia City in May 2024. Then, their petals were separated and kept in a refrigerator at a temperature of 4 ± 1 °C until the experiment. To determine the initial moisture content, 5 samples each 30 g were dried in an oven at 70 °C for 12 h^3^. The initial moisture content of the rose flowers was approximately 78 ± 2% (w.b.). In this research, after picking and separating the petals from other parts of the flower, the rose samples were placed in RW, IR, and CV dryers with different drying temperatures at three levels of 50, 60 and 70 ± 2 °C in the form of a thin layer and dried. In each test, 80 g of fresh samples (in three replicates) were used. The drying of the rose flower continued until it reached a moisture content of about 10% (w.b.). The changes in the weight of the samples during the RW, IR, and CV methods were measured at 5-min intervals to estimate the moisture content. After the samples’ moisture reached the desired size, the dried samples were placed in thick nylon bags, and their lids were tightly closed to prevent moisture absorption from the environment.

### Used laboratory dryers

#### Refractance window

**Fig. 1:**
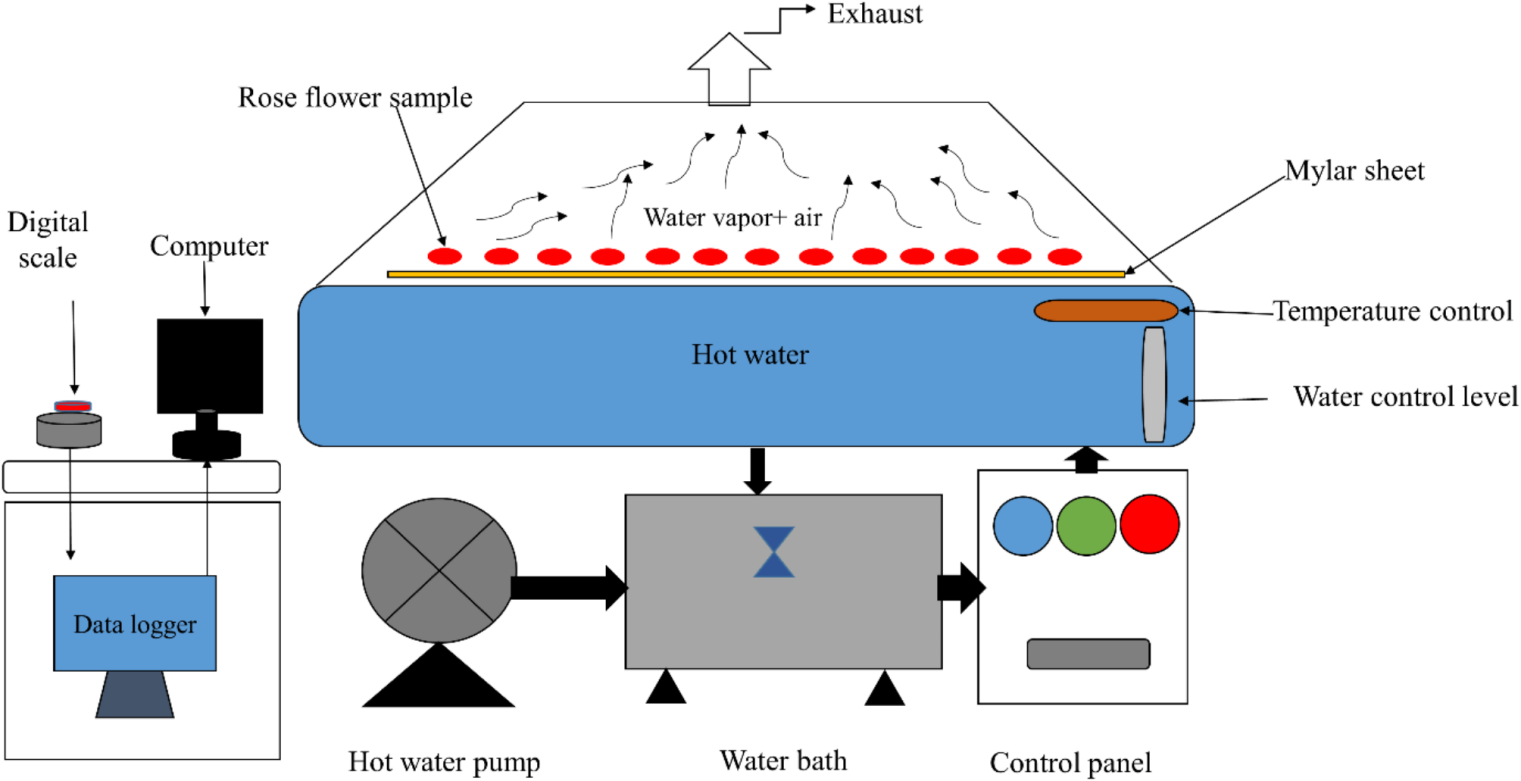
The schematic diagram of refractance window dryer.

In this research, a water bath technology with temperature regulation (RW dryer) was used on a laboratory scale (Fig. 1). In this method, hot water was heated to the desired temperature in a thermostatic water bath with a capacity of 100 L. In addition, hot water was circulated through a steel tank using a centrifugal water pump. A food-grade polyester film (Mylar™, 0.26 mm thick) was placed on the surface of hot water; the rose samples were spread in a single layer and uniformly on the Mylar™ screen. A cap was placed on top of the Mylar™ plate (drying area) to prevent the heat generated from escaping. An industrial electric fan was connected to the top of the chamber to exhaust the water vapors created during the drying process of the roses.

#### Convective dryer

**Fig. 2:**
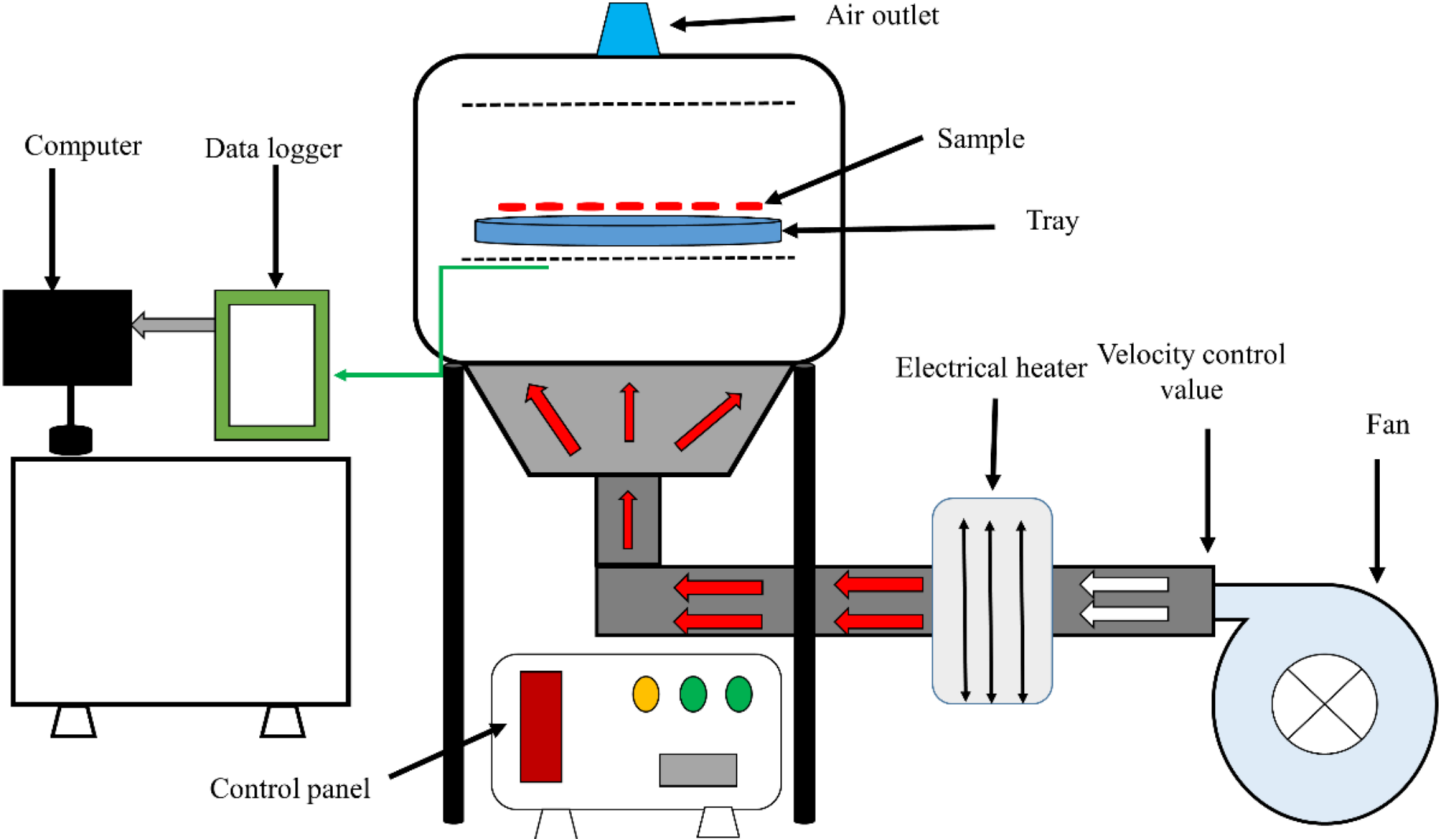
The schematic diagram of the convective dryer.

A laboratory CV dryer (Fig. 2) was used in this research. This device can accurately adjust the drying air temperature between 30 and 110 °C. The laboratory dryer comprises an airflow velocity regulation system, an electric heating part (heater), an air temperature control system, an electric blower, sensors, and a dryer chamber. The heating system of this device included an electric heater installed inside the channel. The temperature was controlled through a control system based on microcontrollers. The samples were weighed during the drying process using a digital scale with a sensitivity of 0.01 g. During the experiments, the air velocity entering the drying chamber was constant and equal to 1 m/s.

#### Infrared dryer

**Fig. 3:**
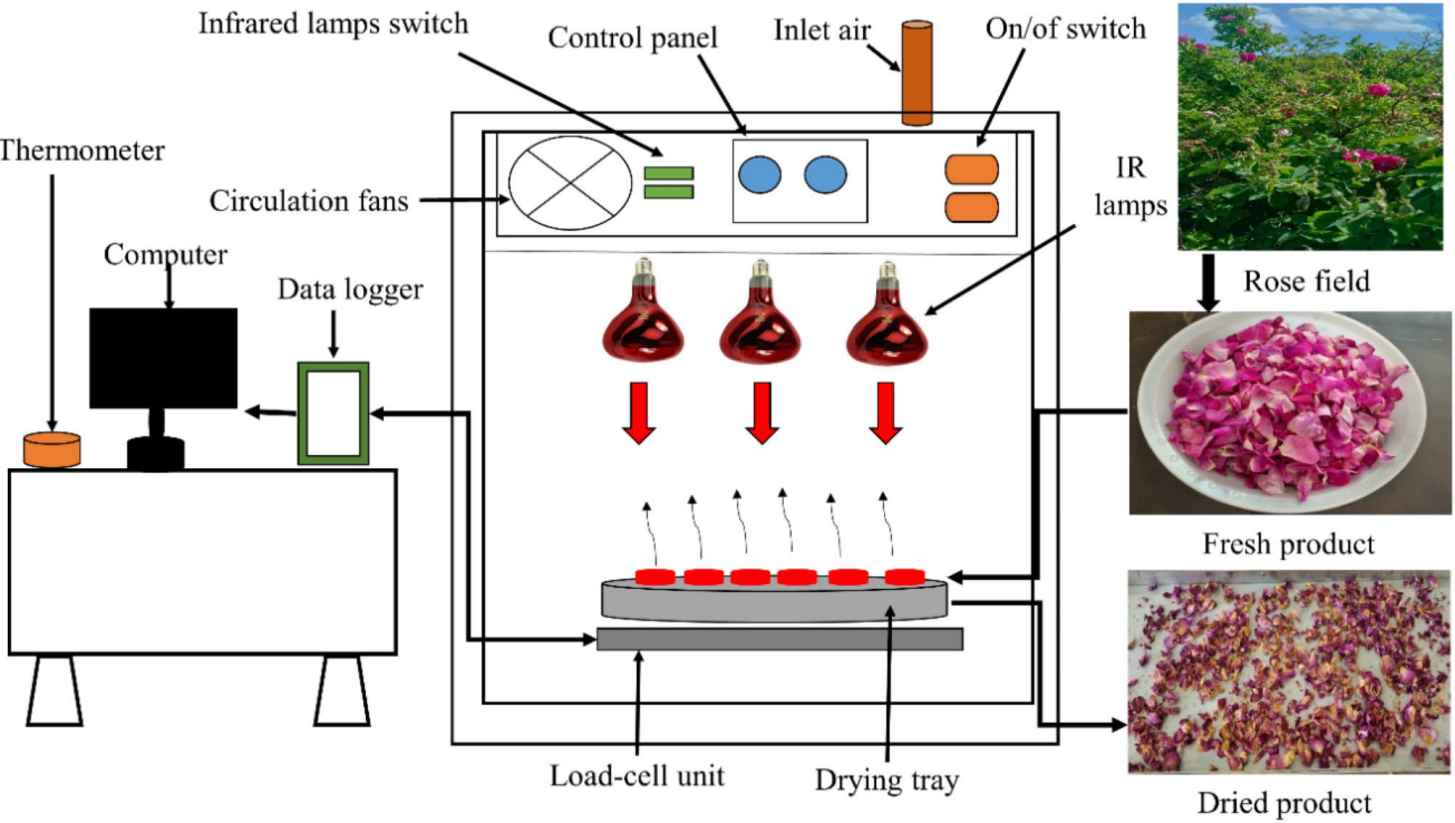
The schematic diagram of the infrared dryer.

In this research, an infrared dryer (Fig. 3) was used to dry the samples of roses. They were placed inside the device as a thin layer. The dryer includes four IR lamps with a distance of 10 cm from the surface of the samples, a drain pipe to remove moisture, a tray for the sample to be placed on it, an electric fan, a control panel, a switch to turn off the IR lamp, and a microcontroller to adjust the drying temperature and a digital scale.

### Moisture ratio

The moisture ratio is important in controlling the drying process. This parameter expresses the moisture content of the rose at each moment, relative to the initial and final moisture, so the moisture content of the samples after drying was calculated using Eq. (1)^5^.

1 $$MR = \frac{{M_{t} - M_{e} }}{{M_{o} - M_{e} }}$$

### Effective moisture diffusivity and activation energy

Fick’s second law was used to calculate the D_eff_^24^.

2 $$\frac{\partial x}{{\partial t}} = D_{eff} \frac{{\partial^{2} x}}{{\partial x^{2} }}$$

Fick’s second law is often used to describe the phenomenon of moisture penetration. The solution of Fick’s equation for a blade is as follows^10^:

3 $$MR = \frac{8}{{\pi^{2} }}\exp \left( { - \frac{{\pi^{2} D_{eff} t}}{{4L^{2} }}} \right)$$

According to the Arrhenius equation, the activation energy (Ea) during the descending drying stage is determined as follows^25,26^.

4 $$D_{eff} = D_{o} \exp \left( {\frac{{E_{a} }}{RT}} \right)$$

### Thermodynamics

The thermodynamic properties of the process of drying roses with three methods RW, IR and CV, taking into account the global gas constant (R = 8.314 J/mol K) using the relationships (ΔH—enthalpy-Eq. 5), (ΔS—entropy-Eq. 6) and (ΔG—Gibbs free energy-Eq. 7) were calculated^27^.

5 $$\Delta {\rm H} = E_{a} - RT$$

6 $$\Delta S = R\left[ {\ln (D_{0} ) - \ln \left( {\frac{{K_{b} }}{{h_{p} }}} \right) - \ln (T)} \right]$$

7 $$\Delta G = \Delta {\rm H} - T\Delta S$$

### Specific energy consumption (SEC)

This energy parameter refers to the ratio of the total energy consumed during the drying process of roses to the amount of water lost during the drying process. It is calculated from Eq. (8)^28^:

8 $$SEC\,({\text{kWh}}/{\text{kg}}) = \frac{{{\text{ Total}}\,{\text{energy}}\,{\text{supllied}}\,{\text{in}}\,{\text{drying}}\,{\text{(kWh)}}}}{{{\text{ Quantity}}\,{\text{of}}\,{\text{moisture}}\,{\text{removed}}\,{\text{during}}\,{\text{drying}}\,{\text{(kg)}}}}$$

### Energy efficiency

This parameter is the ratio of evaporation energy to consumed energy and can be calculated from the following equation^21^.

9 $$EE(\% ) = \frac{{{\text{ E}}_{{\text{e}}} \,{\text{(KW)}}}}{{{\text{ Input}}\,{\text{energy}}\,{\text{(kW)}}}} \times 100$$

10 $$E_{e} \,({\text{KW}}) = Q*h_{fg}$$

11 $$Q = \frac{M}{t}$$

### Quality evaluation

#### Color

To determine the amount of color changes of the dried product and compare it with fresh samples, the method of measuring the total color index changes from the CIE model and the colorimeter device (model CR 400 made by Konica Minolta, Japan) was used. Equation (12) determined the total color changes^29^.

12 $$\Delta E = \sqrt {(L_{0}^{*} - L^{ * } )^{2} + (a_{0}^{*} - a^{ * } )^{2} + (b_{0}^{*} - b^{ * } )^{2} }$$

#### Rehydration ratio

To calculate the rehydration percentage (RR), the dried samples were weighed, then placed in a water bath at 50 °C. After 120 min, the samples were removed from the water and weighed^30^. The rehydration ratio of the rose samples was calculated and reported by Eq. (13)^31^.

13 $$RR = \frac{{{\text{ Weight}}\,{\text{of}}\,{\text{rehydrated}}\,{\text{samples}}\,{\text{(g)}}}}{{{\text{ Weight}}\,{\text{of}}\,{\text{dried}}\,{\text{samples}}\,{\text{(g)}}}}$$

#### Extraction of rose samples essence

In order to extract the essence, 5g from the dried rose flower sample by RW, IR and CV dryers at drying temperatures of 50, 60 and 70 °C were homogenized with 15 cc of methanol for 5 min. Then homogenized at 760 rpm were centrifuged for 10 min; to remove scum and insoluble substances, the supernatant essence was filtered with Whatman paper. The obtained essences were kept in a freezer at − 20 °C until the test.

#### Total phenol content (TPC)

The amount of total phenol was measured by spectrophotometric method and Folin-Ciocaltiu test with a slight change according to the method of Subrahmanyam et al.^31^. To extract phenol, 20 µl of the filtered essence were added to the tube with 180 µl of distilled water. Then 1200 µl of Folin-Ciocalthio reagent (diluted 1:10 with distilled water) were added to the tube. After 3 min of leaving the solution at room temperature (for the reaction between the reagent and the phenolic compound), 2 mL of 7% sodium carbonate solution was added to the mixture. The resulting solution was kept at room temperature for 60 min. Then its absorption value was read using an ultraviolet spectrophotometer (T-80, UV/VIS double beam, Leicester, UK) at a wavelength of 765 nm.

#### Total flavonoid content (TFC)

The TFC was determined based on the aluminum chloride colorimetric method. In this method, 25 µL of essential oil with 75 µL of 5% sodium nitrite was mixed, and after six minutes, 150 µL of 10% aluminum chloride was added to it. After five minutes, 0.5 cc of 1 molar NaOH was added to the mixture and the volume of the solution was brought to 2.5 ml. The amount of absorption in the wavelength of 510 nm was read using an ultraviolet–visible spectrometer (T-80, UV/VIS double beam, Leicester, UK)^32^.

#### Antioxidants activity

The AA of the samples was evaluated by measuring the reduction activity of 2 and 2-diphenyl-1-picrylhydrazyl (DPPH). The anti-radical activity of the rose essence was investigated using the DPPH unstable radical with a slight modification according to the method of Zamani et al.^17^. To perform this test, dilutions of 10 mg/ml and 1 ml were prepared from the samples of primary extracts, then 1 ml of extracts and 0.1 ml of DPPH solution were added to each of the test tubes and mixed well. The amount of light absorbance of the solution after one hour was taken as control light absorbance. At room temperature, we put all the test tubes in the dark for one hour. Then, the optical absorption of the solutions was read by a spectrophotometer at a wavelength of 517 nm. The DPPH radical scavenging activity of the essence, which is a measure of the antioxidant activity of the sample, was calculated according to Eq. (14)^33^:

14 $$AA\,(\% ) = \left( {1 - \frac{{A{}_{sample}}}{{A_{control} }}} \right) \times 100$$

#### Essential oil efficiency

To determine the amount of essential oil, 40 g of dried rose was extracted with the help of a Clevenger machine and distilled with water for 3 h. The amount of water used in each test was 250 ml.

### Statistical analysis

This research was analyzed in a factorial format using a completely random design and SPSS version 21 software. Drying tests were performed in three repetitions, and Duncan’s multi-range test was used at the 95% probability level to compare the average of the observed responses.

## Results and discussions

### Kinetics and drying time

**Fig. 4:**
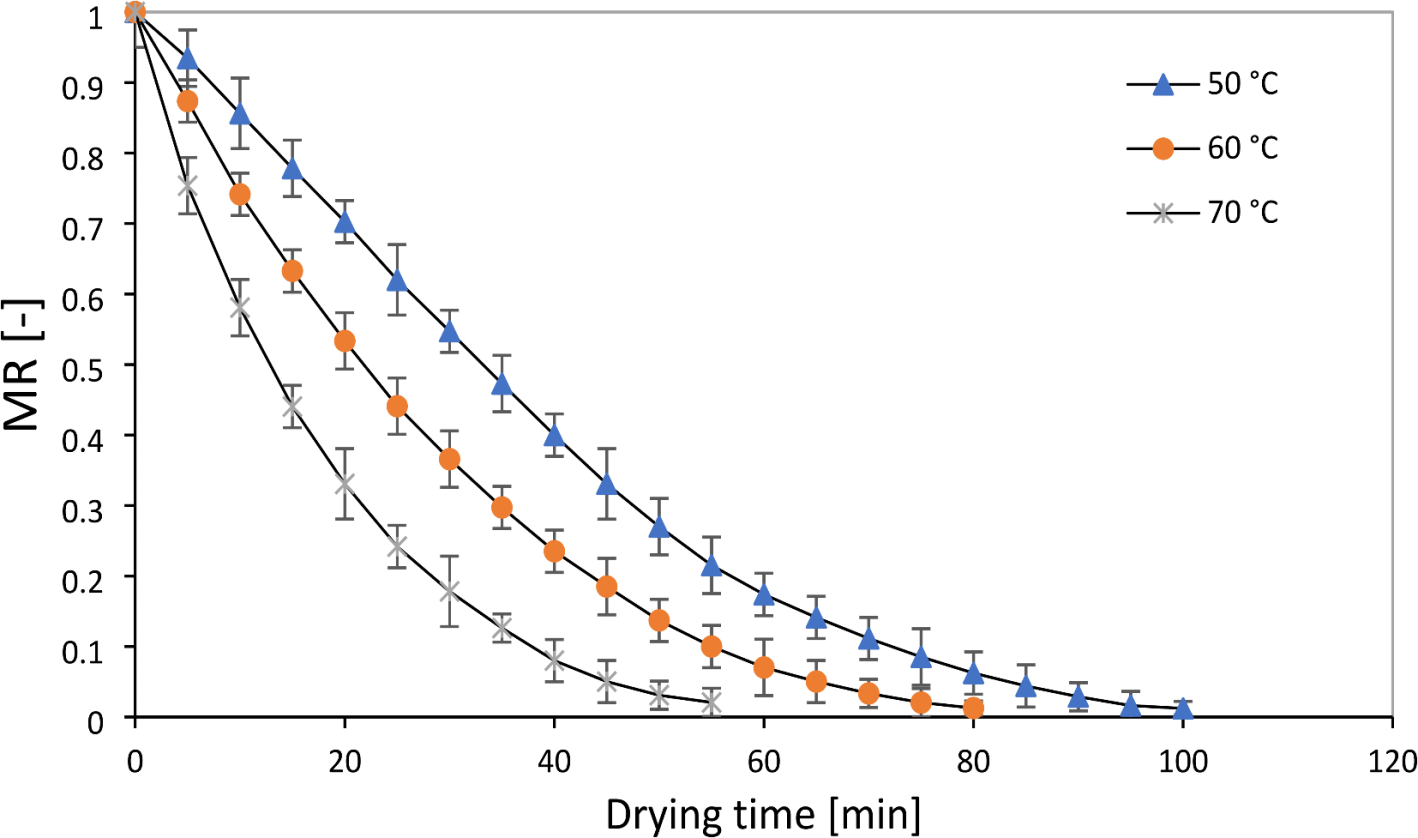
Drying curves of rose flower under refractance window drying processes.

**Fig. 5:**
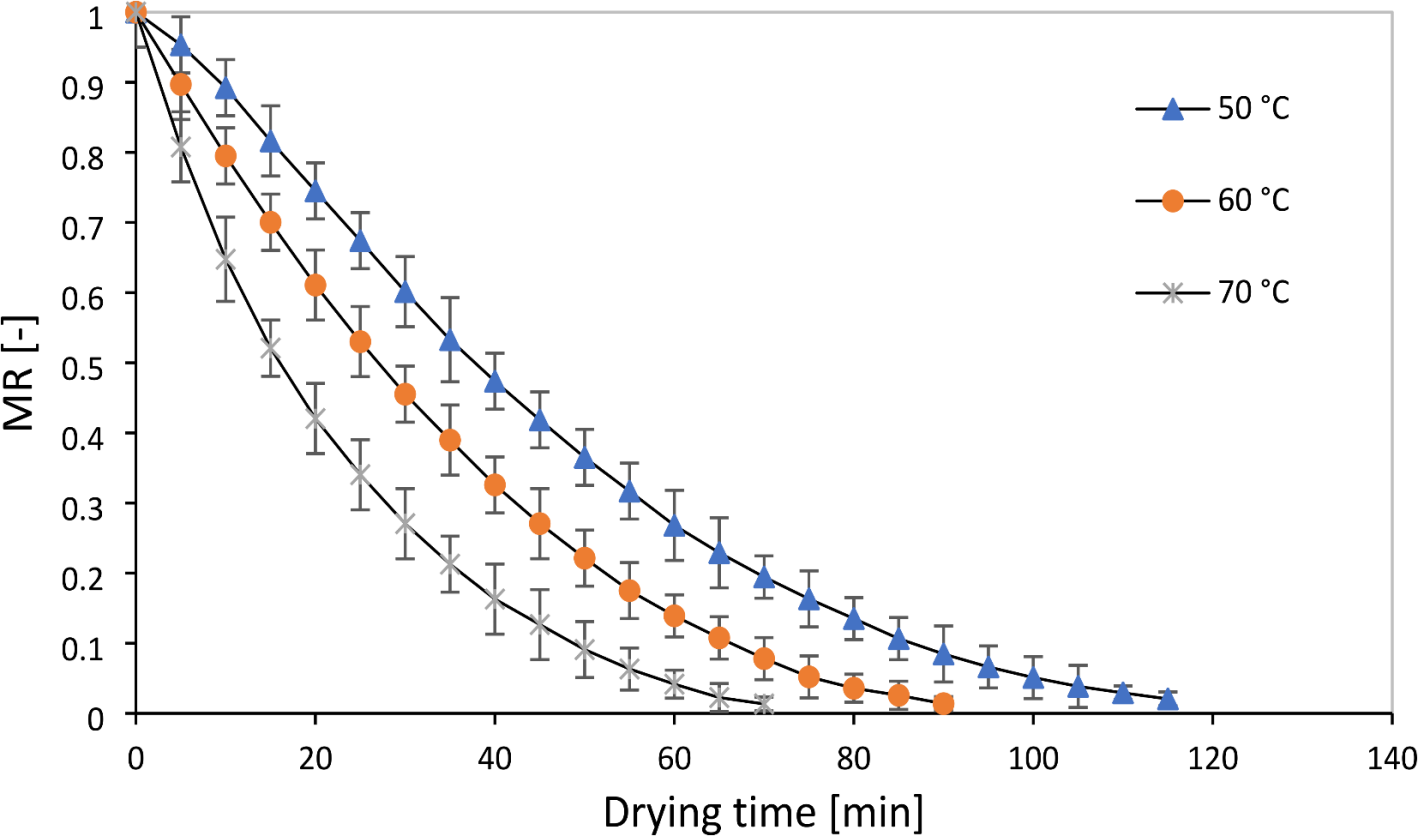
Drying curves of rose flower under infrared drying processes.

**Fig. 6:**
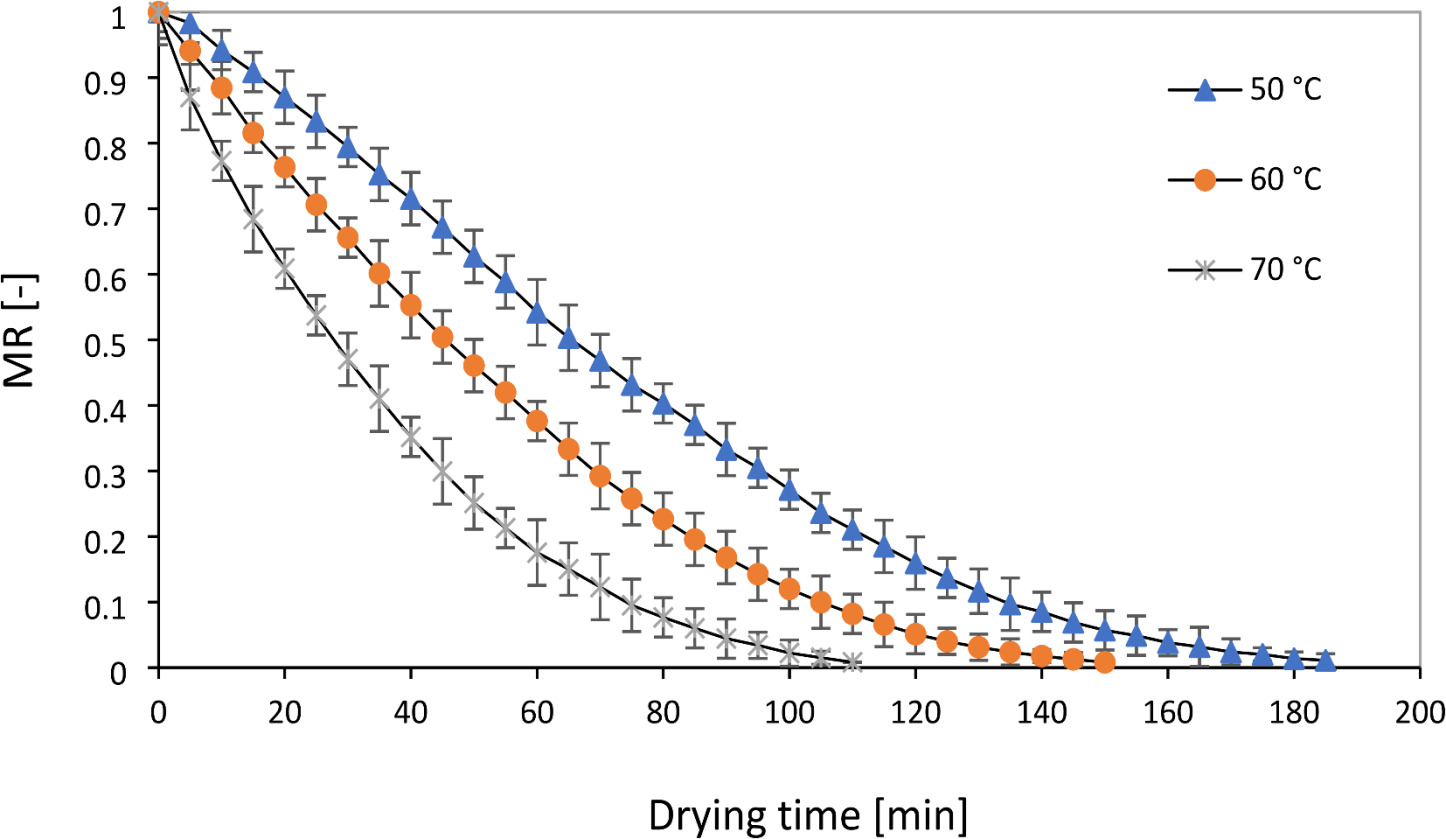
Drying curves of rose flower under convective drying processes.

The initial moisture content of the sample of the leaves (78 ± 2% w.b) decreased to about 10% w.b moisture in the air temperature of 50 to 70 °C. The results of studies indicate that the moisture content of medicinal plants to prevent fungal contamination is 10% based on fresh weight. With the decrease in moisture, the moisture extraction process becomes more difficult, and its cost increases^11^. The diagram of drying kinetics and drying time of the rose samples in RW, IR, and CV methods are shown in Figs. 4, 5, and 6, respectively. As expected, with the increasing acceleration of the drying process of the samples, an exponentially decreasing behavior was observed depending on the temperature. This phenomenon has been reported in different matrices of medicinal plants and different drying technologies^21^. The rate of moisture evaporation is influenced by the movement of water from the inner layers to the surface of plant organs, and the high temperature of dryers plays a significant role in the rapid reduction of moisture content from plant organs, which can reduce the drying time and determine their final quality^33^.

Determining the duration of drying agricultural products is very important. Drying time is a function of plant moisture content and drying temperature. If the amount of plant moisture is less, the plant dries faster, and on the other hand, high drying temperatures accelerate this process by evaporating the plant’s moisture faster. Reducing the drying time of plant products is important in reducing costs related to energy consumption reducing the speed of microbial spoilage and chemical changes^17^. This test showed a significant decrease in drying time due to increasing the drying temperature in all three drying methods (Table 1—*P* < 0.01). Changing the drying temperature from 50 to 60 °C, reduced the drying time in RW, IR and CV methods by about 31.5, 22.2 and 26.90%, respectively, and to 70 °C about 45, 39.10 and 40.5%, respectively. The higher drying temperatures increases the evaporation of moisture in the rose flower samples. It increases the heat transfer rate between the heat source and the product, thus leading to faster removal of moisture and, therefore, reducing the drying time. The reduction of drying time with increasing temperature from 50 to 70 °C for drying physalis in RW method was about 52%^23^, Linden in IR method was about 60%^9^ and saffron petal in the CV method was reported to be about 55.5%^24^.

**Table 1:** Variance analysis for time, D_eff_, SEC, energy efficiency, RR and ΔE of the different dryers and air temperature.

Source	df	Time	D_eff_	SEC	Energy efficiency	RR	ΔE
A—Dryers	2	6808.33**	1.32 × 10^–15^**	53.35**	386.03**	0.73**	77.70**
B—Temperature	2	12433**	1.32 × 10^–15^**	123.42**	438.34**	0.67**	58.63**
A*B	4	233.33**	2.43 × 10^−17ns^	3.48*	88.28**	0.001^ns^	1.25**
Error	16	6.25	1.35 × 10^–17^	0.45	0.06	0.02	1.60**
C.V		2.35	8.25	9.69	2.47	4.94	12.25**

*Significant at *P* < 0.05, **significant at *P* < 0.01, ns not significant at *P* < 0.0.5, CV, coefficient of variation; df, degrees of freedom.

On the other hand, as seen in Table (2), the drying time of the samples in the RW dryer is less than the other two dryers (*P* < 0.01). Because the rate of moisture removal from the samples placed in the RW dryer is higher than that of the other two dryers. RW method significantly reduced the drying time of bananas compared to CV, vacuum, and freezer methods^11^. Approximately 11.1 to 21.4 and 45.90 to 50% reduction in process time was observed for RW drying compared to IR and CV, respectively. Rajoriya et al.^34^ calculated that RW can reduce drying time by 60% compared to CV. It has been reported that RW can make the structure of samples porous due to heat transfer by conduction and radiation and lead to faster diffusion of moisture and reduction of drying time. In this context, similar results have been reported on aloe vera^35^, coriander^33^, and dragon^36^. Therefore, the results of comparing the averages indicate that the highest drying speed occurred at the drying temperature of 70 °C in the RW method, and the lowest was related to the cv treatment at 50 °C (Table 2—*P* < 0.01).

**Table 2:** Values of drying time, D_eff_, SEC and Energy efficiency rose flower at different drying temperatures and dryers.

Dryers	T (°C)	Drying time (min)	D_eff_ (m^2^/s)	SEC (kWh/kg)	Energy efficiency (%)
RW	50	100 ± 5.77 ^e^	4.51 × 10^–8^ ± 2.43 × 10^–9^ ^cd^	4.82 ± 0.28 ^e^	7.14 ± 0.44 ^a^
	60	80 ± 4.61 ^g^	4.99 × 10^–8^ ± 1.67 × 10^–9^ ^c^	3.21 ± 0.30 ^f^	14.32 ± 0.49 ^b^
	70	55 ± 5.77 ^i^	7.05 × 10^–8^ ± 3.52 × 10^–9^ ^a^	2.01 ± 0.24 ^g^	31.28 ± 0.66 ^c^
IR	50	115 ± 5.77 ^c^	3.47 × 10^–8^ ± 1.24 × 10^–9^ ^e^	9.16 ± 0.41 ^b^	4.00 ± 0.46 ^b^
	60	90 ± 3.46 ^f^	4.69 × 10^–8^ ± 1.65 × 10^–9^ ^cd^	6.33 ± 0.29 ^d^	9.73 ± 0.33 ^c^
	70	70 ± 3.46 ^h^	6.07 × 10^–8^ ± 1.65 × 10^–9^ ^b^	4.50 ± 0.29 ^e^	15.05 ± 0.33 ^d^
CV	50	185 ± 2.88 ^a^	2.11 × 10^–8^ ± 1.67 × 10^–9^ ^f^	14.56 ± 0.55 ^a^	1.95 ± 0.13 ^c^
	60	150 ± 2.30 ^b^	3.12 × 10^–8^ ± 2.81 × 10^–9^ ^e^	10.10 ± 0.46 ^b^	3.46 ± 0.30 ^d^
	70	110 ± 2.30 ^d^	4.19 × 10^–8^ ± 2.43 × 10^–9^ ^d^	7.54 ± 0.32 ^c^	5.63 ± 0.36 ^e^

T, Temperature.

### Deff

Table 2 shows the changes in D_eff_ calculated from the Fick model for drying roses in three dryers: RW, IR and CV. As can be seen in the Table 2, the D_eff_ values for rose in RW, IR and CV dryers are 4.50 × 10^–8^ to 7.05 × 10^–8^ m^2^/s, 3.45 × 10^–8^ to 6.09 × 10^–8^ m^2^/s, 2.12 × 10^–8^ to 4.17 × 10^–8^ m^2^/s, respectively. All the D_eff_ values for the three dryers were in the range (10^–6^ to 10^–12^ m^2^/s) reported for agricultural products. Aradwad et al.^3^ reported the D_eff_ value for drying rose in CV was 9.37 × 10^–9^ to 2.16 × 10^–8^ m^2^/s. In addition, they obtained D_eff_ values as 1.73 × 10^–8^ to 4.14 × 10^–8^ m^2^/s during drying of roses in the IR method. According to the reported results, the D_eff_ of rose in the RW dryer is higher than the other two dryers. In the literature, Kırmızıkaya and Doğan^22^ showed that the drying D_eff_ value of Maraş green pepper in RW method is higher than CV and Fluidized bed. Rajoriya et al.^34^ reached similar results. They stated that conductive and radiant heat transfer in the RW method increases the water vapor pressure in the product’s pores and causes changes in the cell structure. Therefore, more homogeneous diffusion occurs in the product’s texture, and water molecules move faster as the drying shortens. Also, D_eff_ is at its lowest value in the CV method compared to the IR method, and the reason for that is the slower drying speed. Among all the conditions studied, drying with CV at a drying temperature of 50 °C obtained the lowest D_eff_ (2.12 × 10^–8^ m^2^/s), while drying with RW at a drying temperature of 70°C had the highest value (7.05 × 10^–8^ m^2^/s). In addition, when comparing the D_eff_ values at different temperatures for each of the drying methods, it is observed that with the increase in temperature, the D_eff_ values tend to increase because the increase in the drying temperature causes the mobility of water molecules, which can affect the increase water transfer^29^. An increase in D_eff_ when increasing temperature was also observed in previous works during RW drying for coriander^33^, mint^37^ and physalis^23^, during CV drying for peppermint leaves^38^ and mint leaves^39^, during IR drying of stevia leaves^40^.

### Activation energy

The E_a_ of rose petals in RW, IR, and CV dryers was 17.88, 26.13, and 31.29 kJ/mol, respectively (Table 3). Compared to CV and IR drying, less E_a_ is required in RW drying. The energy required for water transfer during RW drying of roses was lower as compared to IR and CV drying. Furthermore, diffusion during CV drying takes longer and consumes more energy to occur, while the lowest time and energy requirements were achieved during RW drying^41^. In the previous studies on the drying of roses, the value of E_a_ for the CV drying method was 51.09 kJ/mol, and in the IR method, the value of E_a_ was reported as 25.18 kJ/mol^3^. However, there is no study on E_a_ of roses in RW dryer. But the E_a_ of some medicinal plants such as *Curcuma longa* L and ginger, was obtained by the RW method at 47.05 kJ/mol^10^ and 26.54 to 28.92 kJ/mol^42^, respectively.

**Table 3:** Effects of different drying methods and air temperature levels on thermodynamic parameters for dried rose flower.

Dryers	T (°C)	E_a_ (kJ/mol)	R^2^	ΔΗ (kJ/mol)	ΔS (J/mol K)	ΔG (kJ/mol)
RW	50	17.88	0.9610	15.19 ± 0.03 ^g^	− 154.37 ± 3.77 ^c^	65.08 ± 0.25 ^e^
	60			15.11 ± 0.02 ^h^	− 156.29 ± 4.04 ^c^	67.19 ± .018 ^c^
	70			15.02 ± 0.01 ^i^	− 158.12 ± 4.89 ^c^	69.26 ± 0.31 ^a^
IR	50	26.13	0.9991	23.44 ± 0.01 ^d^	− 125.66 ± 3.52 ^b^	64.06 ± 0.35 ^f^
	60			23.36 ± 0.01 ^e^	− 128.44 ± 2.71 ^b^	66.11 ± 0.38 ^d^
	70			23.27 ± 0.1 ^f^	− 130.92 ± 2.71 ^b^	68.18 ± 0.42 ^b^
CV	50	31.29	0.9952	28.60 ± 0.01 ^a^	− 108.26 ± 3.63 ^a^	63.58 ± 0.26 ^f^
	60			28.52 ± 0.01 ^b^	− 111.38 ± 3.33 ^a^	65.62 ± 0.16 ^de^
	70			28.43 ± 0.01 ^c^	− 114.44 ± 2.92 ^a^	67.68 ± 0.24 ^bc^

### Thermodynamic properties

Table 4 shows the effects of temperature and dryer on thermodynamic (ΔH, ΔS and ΔG) properties. Drying temperature and type of dryer have a significant impact on thermodynamic (ΔH and ΔG) properties. However, the type of dryer has no considerable effect on ΔS. The enthalpy values (ΔH) that define the energy changes between the activated complex and the reactant are shown in Table 3. Table 3 shows that the obtained ΔH values were 15.15–19.03, 23.23–44.28 and 28.28–44.28 kJ/mol for the RW, IR and CV drying systems, respectively. Its highest value was observed in the CV dryer at a temperature of 50 °C and the lowest value in the RW dryer at a drying temperature of 70 °C. The positive figures from the enthalpy changes in all three dryers show that the endothermic reaction occurred. From Eq. (5), the change in ΔH can be attributed to the drying temperature, and as can be seen, the enthalpy value decreases in all three drying methods as the temperature increases. A similar process was used by Rashid et al.^43^, Akhoundzadeh Yamchi et al.^5^ and Cunha et al.^10^ for drying sweet potatoes (by CV and IR methods), truffle (by IR method) and *Curcuma longa* L (by RW method), respectively. The enthalpy value in the CV method was 18% higher than that of IR and 47% higher than that of the RW method, while enthalpy in the IR method was almost 35% higher than that of RW. Lower enthalpy values correlate with less energy required to initiate mass transfer during drying.

**Table 4:** ANOVA results of different air temperatures and dryers on thermodynamic attributes of rose flower.

Source	df	ΔΗ (kJ/mol)	ΔS (J/mol K)	ΔG (kJ/mol)
A—Dryers	2	0.062**	57.66^ns^	38.47**
B—Temperature	2	411.77**	4626.39**	5.63**
A*B	4	0^ns^	1.13^ns^	0.001^ns^
Error	16	0.0001	39.82	0.24
C.V		0.05	− 4.78	0.75

*Significant at *P* < 0.05, **significant at *P* < 0.01, ^ns^ not significant at *P* < 0.0.5, CV = Coefficient of variation, df = Degrees of freedom.

Entropy change (ΔS) was also evaluated for the disordered change in the movement of molecules between moisture and rose flower. Table 4 shows that ΔS values were negative in all drying systems. The obtained value ranges from − 158.10 to − 154.37 J/mol K for the RW method, from − 130.90 to − 125.69 J/mol K for the IR method, and from − 114.37 to J/mol K to − 108/23 J/mol K for the CV method. Negative entropy values occur due to changes in the structure of molecules or chemical properties during the drying process. The entropy value decreased as the temperature increased from 50 to 70 °C in all three methods (Table 4—*P* < 0.01). Due to the increased excitation of water molecules, less entropy was obtained at high-temperature levels and vice versa. Similar reports of entropy decrease with increasing temperature have been presented by Cunha et al.^10^.

The values of Gibbs free energy during drying of roses are positive for all drying temperatures in all three methods; these positive values indicate an endothermic drying process. The values of ΔG for RW, IR and CV dryers were from 65.08 to 69.28, 64.06 to 68.19, and 63.57 to 67.68 kJ/mol, respectively, which indicate non-spontaneous reactions. Therefore, thermal energy is required to initiate and maintain drying from the dryer system. According to the study of El-Mesery et al.^44^, the value of ΔG for drying garlic in CV and IR-CV methods was between 222 and 235.4 and 218.9 and 232.4 kJ/mol, respectively. Also, in another study, Chouaibi et al.^45^ reported the value of ΔG to obtain ascorbic acid during eggplant drying in the range of 83.16–87.13 and 84.82–86.80 kJ/mol, respectively, for CV and IR methods. As can be seen from Table 4, the highest value of ΔG was obtained in the RW method and at a drying temperature of 70 °C, while the lowest value was obtained in the CV method and at a temperature of 50 °C (*P* < 0.01). Also, using the RW method compared to the IR and CV methods increases the value of ΔG by 3% and 1.5%, respectively. A similar trend was observed by Santos et al.^46^ in the RW method, de Jesus Junqueira et al.^25^ in the IR method, and de Vilela Silva et al.^27^ in the CV method.

### Special energy consumption

Food drying is considered one of the most energy-intensive processes. The energy consumption per kilogram of water removal from rose samples for different drying methods (RW, IR, and CV) is presented in Table 2. According to the ANOVA (Table 1), drying temperature and dryer had a significant effect on SEC (*P* < 0.01). Significantly, RW reduced SEC by 66.80–73.30% and 47.2–57.2%, respectively, compared to CV and IR (*P* < 0.01). In addition, the use of IR reduced SEC by 37.2–40.3% compared to CV. Likewise, Rajoriya et al.^34^ stated that the RW method reduced the SEC of apple drying by 18–20% compared to CV and attributed it to a significant reduction in drying time. Also, Seyfi et al.^35^ showed that the RW method’s SEC was lower than CV’s. Increasing the temperature in RW, IR, and CV from 50 to 70 °C reduced SEC by 58.3%, 50.9%, and 48.35%, respectively. Among the studied treatments, the lowest amount of SEC was observed in the RW method (2.01 kWh/kg) at the drying temperature of 70 °C (*P* < 0.01).

### Energy efficiency

Drying temperature and dryer significantly affected drying efficiency (Table 1—*P* < 0.01). The comparison of different modes in the Table 2 shows that increasing the temperature from 50 to 70 °C in all three dryers (RW, IR and CV) increases the energy efficiency. Temperature increases the moisture removal rate from the product, reduces the drying time, and increases energy efficiency (*P* < 0.01). This result is similar to the results obtained from drying aloe vera^35^, in which energy efficiency is said to increase with increasing temperature. On the other hand, by comparing different dryers, it can be found that the highest energy efficiency values are when the RW dryer is used at a drying temperature of 70 °C, and the lowest efficiency of the CV method is seen at 50 °C. The research results show that the RW method increases energy efficiency compared to the IR and CV methods. The reason for this could be the increase in moisture removal speed in the RW method compared to the other two methods. RW prevents the formation of a hard layer in the product, increasing moisture removal and energy efficiency. Baeghbali et al.^12^ showed that the energy efficiency of RW dryer was higher compared to spray and freeze. In another study, Baeghbali et al.^15^ who dried apples using different methods concluded that the energy efficiency of RW was higher than that of CV and freeze.

### Color

Color is the most important quality feature that affects consumer acceptance of a product. During the heat treatment of fruits, herbs, and vegetables, L*, a*, and *b is widely used to describe the color change. The drying temperature and drying method significantly affected the color parameters of dried rose flowers (Table 5—*P* < 0.01). Table 6 shows the color value of dried rose flowers. As seen in Table 6, the values of fresh roses’ L*, a*, and b* were 40.77 ± 0.73, 14.22 ± 0.21, and 1.66 ± 0.1, respectively. These values were obtained after drying with the RW method as 32.73 to 36.65, 16.25 to 19.35, 3.62 to 5.05, respectively, with the IR method as 31.61 to 34.75, 17. 26 to 23.24, 2.94 to 4.79, respectively, and by CV method as 29.28 to 32.65, 19.21 to 25.11, 2.40 to 3.47, respectively. The results found that compared to the fresh samples, L* values significantly decreased during drying (in all three methods), but a* and b* values increased. This may be because the color of the sample is blacker, redder, and yellower than the color of the fresh rose. Hnin et al.^47^ obtained similar results in drying roses at 50 to 70 °C temperatures in an infrared-freeze dryer. They showed that the L*, a*, and *b values of roses after drying were between 34.6 to 36.3, 23.5 to 24.3, and − 3 to − 3.4, respectively. Also, Zalpouri et al.^33^ reported that with increasing drying temperature in the RW method for drying coriander, the value of L* index decreases and the value of a* increases. The amount of L* of dried rose flowers was lower than that of fresh flowers due to the browning caused by the drying process. Also, as the temperature increases, the value of L* decreases in all three methods. On the other hand, the values of redness (a∗) and yellowness (b*) increased with increasing temperature in all three processes of RW, IR and CV. According to the results, the values of a* (redness) of roses dried with heat increased significantly compared to the fresh sample. The high redness may be related to the degradation of the pigment during the drying process.

**Table 5:** ANOVA results of different air temperatures and dryers on color attributes of rose flower.

Source	df	L*	a*	b*
A—Dryers	3	73.64**	62.43**	6.91**
B—Temperature	2	27.29**	56.33**	4.80**
A*B	4	0.90^ns^	2.42^ns^	2.34^ns^
Error	18	1.14	1.15	0.076
C.V		3.1	5.4	8.01

*Significant at *P* < 0.05, **significant at *P* < 0.01, ^ns^ not significant at *P* < 0.0.5, CV = Coefficient of variation, df = Degrees of freedom.

**Table 6:** Effect of the drying method and drying temperature on the color value (L^*^, a^*^, and b^*^) and rehydration ratio of rose flower.

Dryers	T (°C)	L*	a*	b*	ΔE	RR
Fresh	–	40.77 ± 0.73 ^a^	14.22 ± 0.21 ^f^	1.66 ± 0.10 ^f^	–	–
RW	50	36.65 ± 0.79 ^b^	16.25 ± 0.69 ^e^	3.62 ± 0.16 ^c^	5.31 ± 1.27 ^e^	3.00 ± 0.09 ^bc^
	60	35.20 ± 0.70 ^b^	17.59 ± 0.81 ^de^	4.42 ± 0.14 ^b^	7.28 ± 1.38 ^e^	3.28 ± 0.08 ^b^
	70	32.73 ± 0.43 ^c^	19.35 ± 0.71 ^d^	5.05 ± 0.17 ^a^	10.32 ± 0.99 ^cd^	3.54 ± 0.07 ^a^
IR	50	34.75 ± 0.68 ^b^	17.26 ± 0.64 ^e^	2.94 ± 0.14 ^d^	7.14 ± 0.43 ^e^	2.60 ± 0.06 ^de^
	60	32.39 ± 0.45 ^c^	21.25 ± 0.68 ^c^	3.44 ± 0.17 ^c^	11.27 ± 0.50 ^bcd^	2.92 ± 0.08 ^c^
	70	31.61 ± 0.42 ^c^	23.24 ± 0.65 ^b^	4.79 ± 0.18 ^ab^	13.40 ± 0.52 ^b^	3.21 ± 0.09 ^b^
CV	50	32.65 ± 0.49 ^c^	19.21 ± 0.51 ^d^	2.40 ± 0.17 ^e^	9.75 ± 1.12 ^d^	2.44 ± 0.05 ^e^
	60	31.87 ± 0.69 ^c^	22.24 ± 0.59 ^bc^	2.74 ± 0.15 ^de^	12.30 ± 0.60 ^bc^	2.76 ± 0.10 ^cd^
	70	29.28 ± 0.50 ^d^	25.11 ± 0.76 ^a^	3.47 ± 0.13 ^c^	16.10 ± 0.56 ^a^	3.01 ± 0.05 ^bc^

The L* value of RW samples was slightly lower than IR and RW samples in other treatments, except at the drying temperature of 70 °C. At the same drying temperature, the RW sample’s redness value (a*) was significantly lower than that of IR and CV flowers. However, the CV sample was redder than IR and RW. This shows that the color of roses becomes brighter and redder after drying with CV compared to RW and IR. The redness value (a*) of dried roses may be due to a browning reaction during drying. According to the average comparison results in Table 6, the most color changes were obtained in the CV dryer and the least in the RW method (Table 1—*P* < 0.01). Leiton-Ramírez et al.^48^ reported that RW preserves the color of paprika and guava, respectively, better than CV drying due to less oxidation and degradation of pigments.

### Rehydration ratio

The RR indicates the damage to the internal cell structure and is a quality indicator in the processing of rose samples under different drying conditions of RW, IR, and CV. The results of RR of rose samples at different drying temperatures for RW, IR, and CV methods are summarized in Table 6. Rehydration ratios in all three methods at 70°C were significantly higher than 60 °C and 50 °C (*P* < 0.01). High temperatures, by affecting the cell structures and texture of the product, lead to its loosening and creating a porous structure in the samples, thus facilitating water reabsorption in the created cavities^49^. Similarly, a similar trend was observed for mint leaves^39^ in the CV method, aloe vera^50^ in the RW method, and bitter melon^51^ in the IR method.

For roses dried by RW, IR, and CV, the RR varied from 3.00 ± 0.09 to 3.54 ± 0.08, 2.60 ± 0.06 to 3.21 ± 0.08, and from 2.44 ± 0.05 to 3.01 ± 0.05, respectively. In these conditions, the lowest value related to CV dryer and temperature of 50 °C and the highest value of RR occurred in RW dryer and temperature of 70 °C (*P* < 0.01). It was also observed that at constant drying temperatures, the value of RR using RW was significantly higher than that of IR and CV samples (*P* < 0.01). This was probably because RW has preserved the internal cellular structure of the rose specimens well. Baeghbali et al.^15^ reported that the RR value of dried apple samples in RW method was higher than CV. In addition, the average RR in IR drying at all three temperatures was higher than CV drying (*P* < 0.01). The surface capillaries caused by the long drying time in the CV method create structures with lower and more uneven pores, which caused the RR value to decrease in this method compared to other methods.

### Total phenol content

Phenolic compounds are a large group of plant secondary metabolites that often have antioxidant activity. These compounds are hydrophilic non-enzymatic antioxidants and have valuable antimicrobial, antiviral, anti-mutation, and anti-cancer properties. According to Table 7, drying temperature and type of dryer are significant on TPC (*P* < 0.01). Table 8 shows the average TPC values during roses’ drying using RW, IR and CV at different temperatures. The TPC of rose flowers varies depending on the temperature and drying methods. The results of comparing the averages indicate that measuring the amount of TPC in the tissues of fresh roses was superior to all treatments in all methods (*P* < 0.01). A similar result was reported by Barani et al.^52^ and Hnin et al.^47^ for rose flower drying. But TPC values in RW method at 60 °C (58.67 mg GAE/ g dry matter) were closer to fresh sample values (67.26 mg GAE/ g dry matter) (*P* < 0.01). In the drying method with CV, the amount of TPC showed a noticeable and significant decrease compared to other methods (Table 8). Due to longer processing time, decomposition of phenolic compounds, and direct contact of the heating medium during the drying process in CV technique compared to RW, TPC decreased^11^. It was observed that TPC in drying by RW method was higher than CV and IR by 19.1 to 27.7% and 10.90 to 18.80%, respectively. The reason was the facilitation of the release of bound phenolics from the cell matrix of the dried rose samples due to rapid heating during RW drying. Similarly, Rajoriya et al.^34^ observed a 19% increase in TPC values of dried apple slices with RW compared to CV. Also, the results of Zalpouri et al.^14^ confirmed that the RW method, compared to the CV method, led to better preservation of onion phenolic compounds in the best possible way, which is probably due to the inactivation of enzymes in this method.

**Table 7:** Variance analysis for Total phenol, total flavonoid content, antioxidant activity and essential oil of the different dryers and air temperature.

Source	df	TPC	TFC	AA	EO
A—Dryers	3	466.98**	405.98**	988.20**	0.006**
B—Temperature	2	167.41**	272.37**	319.33**	0.005**
A*B	4	187.03**	2.03^ns^	0.26^ns^	0.00001^ns^
Error	18	3.35^ns^	2.87	10.56	0.0004
C.V		4.3	5.35	4.19	6.81

*Significant at *P* < 0.05, **significant at *P* < 0.01, ^ns^ not significant at *P* < 0.05.

**Table 8:** Total phenol content, total flavonoid content, antioxidant activity and essential oil of different drying techniques at different air temperatures applied to rose flower.

Dryers	Temperature (°C)	TPC (mg GAE/ g d.m)	TFC (mg QE/g d.m)	AA (%)	EO (%)
Fresh	–	67.26 ± 0.32 ^a^	47.34 ± 0.44 ^a^	98.01 ± 0.57 ^a^	1.02 ± 0.01 ^cde^
RW	50	49.58 ± 1.34 ^ed^	27.38 ± 0.88 ^e^	79.01 ± 1.67 ^d^	1.10 ± 0.03 ^a^
	60	58.67 ± 1.75 ^b^	39.80 ± 1.21 ^b^	90.66 ± 1.53 ^b^	1.29 ± 0.04 ^ab^
	70	55.32 ± 1.22 ^bc^	35.70 ± 1.01 ^c^	85.01 ± 2.12 ^c^	1.18 ± 0.05 ^bcd^
IR	50	44.70 ± 1.15 ^f^	24.79 ± 0.81 ^f^	67.99 ± 2.23 ^f^	1.00 ± 0.04 ^bc^
	60	52.33 ± 0.95 ^cd^	34.34 ± 1.00 ^cd^	80.01 ± 2.07 ^cd^	1.14 ± 0.05 ^be^
	70	46.55 ± 0.96 ^ef^	32.53 ± 0.95 ^d^	74.99 ± 2.07 ^de^	1.05 ± 0.04 ^de^
CV	50	40.23 ± 1.07 ^g^	19.24 ± 1.01 ^g^	60.01 ± 1.38 ^g^	0.94 ± 0.03 ^bd^
	60	49.26 ± 1.41 ^ed^	29.21 ± 0.91 ^e^	71.99 ± 1.97 ^ef^	1.06 ± 0.02 ^de^
	70	43.34 ± 1.30 ^fg^	26.36 ± 0.96 ^ef^	66.99 ± 1.66 ^f^	0.99 ± 0.03 ^e^

On the other hand, increasing the temperature from 50 to 60 °C increased the amount of TPC by 18.33, 17.06, and 22.44% in RW, IR, and CV methods, respectively. The low TPC during drying at the drying temperature of 50 °C is due to some physical and chemical changes, such as the partial destruction of various types of phenolic compounds and the slow inactivation of oxidative enzymes such as peroxidases and polyphenol oxidases due to the long drying process^53^. However, increasing the temperature from 60 to 70 °C causes a 5.07, 11.04, and 12.01% decrease in TPC values for RW, IR, and CV techniques, respectively. Therefore, 60 °C treatment in all methods improved the extraction of phenolic compounds compared to other temperatures. Medium drying temperature in drying green banana flour in RW^54^, physalis in RW^23^, jujube in CV^55^, *stevia rebaudiana* leaf in CV^56^ and areca taro in IR^57^ has resulted in preserving the TPC of these plants in the best possible way.

### Total flavonoid content

The results showed that fresh plants and plants dried in the RW method with a temperature of 60 °C showed the highest accumulation of TFC with a numerical value of 47.34 and 39.80 mg quercetin/g dry matter, respectively, and there was a significant difference between the dryer and the drying temperatures (Table7—*P* < 0.01). The samples dried at constant temperature under the CV method accumulated the lowest TFC in their tissues. While the highest value was observed in the RW method (Table 8). This trend was observed in the study conducted by Subrahmanyam et al.^31^, where TFC was higher in RW than other dryer types. In the RW method, heat transfer mainly occurs by IR radiation (Mylar plate) and indirectly. Hence, it helps to evaporate moisture faster and reduce drying time, which allows the preservation of TFC compounds in this sample^58^.

The present study showed that increasing the drying temperature in all three dryers from 50 to 60 °C significantly increased TFC. However, increasing the temperature from 60 to 70 °C significantly decreased TFC (*P* < 0.01). The lower value of TFC in all three drying methods at the drying temperature of 50 °C compared to other drying temperatures due to the inactivation of enzymes is effective in decomposing and destroying flavonoids. At the same time, the drying temperature of 60 °C is also due to the optimum temperature for these enzymes increasing TFC. The report of some researchers shows the positive effect of medium temperature and RW method on TFC of physalis products, so medium temperature provides a higher shelf life of TFC compared to high and low temperatures^23^. Ai et al.^59^ in the CV method and Bassey et al.^60^ in the IR method presented similar reports.

### Antioxidants activity

Based on the results of data variance analysis, the effect of different drying methods and drying temperatures on AA showed a significant impact at the probability level of one percent (Table 7). According to the results of comparing the averages, Fresh roses had a higher percentage of AA. As the significant reduction of antioxidant activity of stevia rebaudiana^61^, rosehip^62^ and edible roses^63^ after drying has been reported in different ways compared to the control (fresh samples). The results showed that AA improved by 26.80% to 31.68% and 13.31% to 16.20% in the RW method compared to CV and IR. Also, AA in the IR dryer increased from 11.14 to 13.31% compared to CV. According to Subrahmanyam et al.^31^, apple drying with RW resulted in significant retention of bioactive compounds compared to CV drying.

Similar results have been reported for spent coffee grounds^19^. Creating direct heat using mylar plate in RW treatment creates favorable conditions for releasing intracellular compounds of plant matter, so the amount of AA increases compared to other treatments. On the other hand, the long time in the CV method not only does not provide an opportunity for the release of compounds with antioxidant properties but also destroys these compounds by creating a slow process for drying. According to the average comparison results in Table 8, increasing the temperature from 50 to 60 °C caused a significant increase in the amount of AA in all three methods. However, with the increase in temperature from 60 to 70 °C, this value decreased significantly. The decrease of AA with increasing temperature from 60 to 70 °C is due to the destructive effects of temperature on phenolic compounds, which directly affect the AA of rose extracts. Also, one of the reasons for the decrease in the amount of phenolic compounds at high temperatures can be attributed to the effect of heat on tannic compounds, destruction of cells, and the reduction of the activity of antioxidant enzymes^35^. Therefore, the lowest amount of antioxidant activity was related to the drying treatment at 50 °C in the CV method. In addition, drying at a drying temperature of 60 °C in all three dryers was superior in increasing AA compared to other temperatures (Table 8).

### Essential oil efficiency

During the drying process, the aromatic and fragrant compounds and water evaporate from the plant organs. As a result, the quality of the dried product decreases due to the loss of some of these compounds. Therefore, choosing the appropriate method of drying medicinal plants depends on the type of organ, the purpose of drying, and the moisture content. According to the analysis of the variance table, the effect of different drying methods and drying temperature on the percentage of essential oil was significant (Table7—*P* < 0.01). The comparison of the averages showed that fresh rose had 1.02% essential oil efficiency content. In contrast, the highest amount of essential oil (1.29%) was found in dried samples at a drying temperature of 60 °C and obtained in the RW dryer (Table 8). The minimum percentage of essential oil is equal to 0.94%, obtained using the CV drying method at 50 °C.

As the temperature increased from 50 to 60 °C, the percentage of essential oil increased significantly. However, a further increase in temperature from 60 to 70 °C slightly reduced the extracted essential oil. Nozad et al.^64^ stated that the reduction of essential oil efficiency at higher temperatures is due to the damage to the oil-producing glands the evaporation of oil molecules and volatile compounds during the drying process. On the other hand, in the study of Mokhtarikhah et al.^65^, it is reported that higher temperatures cause more decay of the cell wall and plasma membrane, and this may affect the permeability of the plasma membrane, thus leading to a decrease in EO. In addition, the amount of essential oil extracted in all dryers was higher at 70 °C than at 50 °C. This can be attributed to the sensitivity of the rose to the drying temperature. As can be seen, the lower temperature (50°C) in all three dryers was the lowest in terms of percentage of essential oil. According to Setareh et al.^66^ some volatile compounds and more oil glands may decrease and damage the plant tissue during long-term drying at lower temperatures. According to the results of this study, some authors showed that increasing the drying temperature first increases and then decreases the essential oil of plants such as spearmint^64^ and lemongrass^66^.

The results of the effect of different drying methods on the efficiency of essential oil extraction are presented in Table 8. The results indicate that the drying method significantly affected EO (Table7—*P* < 0.01). As mentioned in previous studies, drying plants before extraction can increase or decrease the extraction efficiency of effective compounds depending on environmental conditions, glandular trichome structure, genotype, essential oil chemical components, temperature, time and drying method^67^. A comparison of essential oil efficiency in RW, IR, and CV drying showed that RW had more EO efficiency at the same drying temperature than other drying methods and fresh samples. Rapid and mild drying in RW minimizes the degradation and oxidation caused by heat and maximizes the product’s flavor^68^. Consistently, plants dried in CV had lower essential oil content than IR and fresh samples. This phenomenon is probably due to the negative effect of long-term drying conditions on the structure of tuberous trichomes and their gradual degradation^69^.

## Conclusions

Rose flower was dried under drying with 3 techniques (Refractance window, infrared and convective) and showed different results according to drying time, specific energy consumption, quality preservation and improvement of bioactive properties and essential oil extraction. According to the results obtained in this research, the long drying time of the roses using the CV method caused some of the quality characteristics of the final product to drop. In addition to reducing drying time and energy consumption, the use of RW improved the qualitative and bioactive characteristics and efficiency of essential oil and increased its energy efficiency compared to infrared and convective methods. The present research showed that the RW drying technique accelerates the drying process and reduces the drying time and energy consumption by 11.1 to 21.4% and 47.2 to 57.2%, respectively, compared to IR, while the corresponding value for CV was 45.90 to 50% and 66.80 to 73.3%. By increasing the drying temperature in all three methods, the moisture diffusion coefficient, energy efficiency, z-Gibbs energy, rehydration ratio, index a* and b* and total color changes increased and compared to drying time, specific energy consumption, entropy, enthalpy and the L* index decreased. The best drying treatment to maintain the bioactive properties and increase the efficiency of the essential oil in all methods occurred at the drying temperature of 60 °C, while the drying temperature of 70 °C, due to the lowest drying time and specific energy consumption and the highest efficiency energy had more satisfactory results than other temperatures. At a drying temperature of 70 °C compared to 60 °C in all three dryers, some undesirable changes occurred in the samples, such that the rate of degradation of bioactive properties and color changes increased, while the efficiency of essential oil decreased significantly. Also, the drying temperature of 50 °C in all three dryers contributed to prolonging the drying time, reducing the rehydration ratio, essential oil efficiency and energy efficiency, and further destroying the number of antioxidants, phenol, and total flavonoids. From this study, it can be concluded that the drying method can significantly affect the qualitative properties, bioactivity, and efficiency of the essential oil of rose flowers. In addition, this research confirmed that RW drying can be a potential method for handling heat-sensitive products such as medicinal plants to obtain better quality products in a relatively shorter time.

## Data Availability

Data will be made available upon request from the corresponding author.

## List of symbols

AA: Antioxidant activity (%)
A_Sample_: Absorbance values of the blank (–)
A_control_: Absorbance values of the sample (–)
D_eff_: Effective moisture diffusivity (m^2^/s)
Ea: Activation energy (J/mol)
h_p_: Planck constant (6.626 × 10^–^^37^ m^2^ kg/s)
h_fg_: The latent heat of vaporization is equal to 2257 kJ/kg (kJ/kg)
K_b_: Boltzmann constant (1.38 × 10^–^^26^ J/K)
$\text{L}$: The thickness of the sliced cantaloupe (m)
M_e_: Equilibrium moisture contents (% d.b)
MR: Moisture ratio (–)
M: Moisture removed during drying (Kg)
M_o_: Final Moisture content of the sample (% d.b)
M_t_: Moisture content at time t (% d.b)
Q: Represents the moisture removed per unit time (–)
R: Universal gas constant (8.314 J/mol K)
SEC: Specific energy consumption (kWh/kg)
t: Drying time (s)
T: Absolute temperature (K)
UNFCCC: United Nations Convention on Climate Change
ΔH: Enthalpy (J/mol)
ΔG: Gibbs free energy (J/mol)
ΔS: Entropy (J/mol K)
ΔL∗, Δb∗, Δa∗: The difference between the color of fresh and dried samples (–)
